# Indoor Radon Concentration Levels in Healthcare Settings: The Results of an Environmental Monitoring in a Large Italian University Hospital

**DOI:** 10.3390/ijerph20064685

**Published:** 2023-03-07

**Authors:** Luigi De Maria, Stefania Sponselli, Antonio Caputi, Giuseppe Delvecchio, Gianmarco Giannelli, Antonella Pipoli, Francesco Cafaro, Silvia Zagaria, Domenica Cavone, Rodolfo Sardone, Luigi Vimercati

**Affiliations:** 1 Interdisciplinary Department of Medicine, University of Bari, 70124 Bari, Italy; 2 Unit of Data Sciences and Technology Innovation for Population Health, National Institute of Gastroenterology “Saverio de Bellis”, Research Hospital, Castellana Grotte, 70013 Bari, Italy; 3 Unit of Epidemiology and Statistics, Local Healthcare Authority of Taranto, 74121 Taranto, Italy

**Keywords:** radon exposure, healthcare workers, reference levels

## Abstract

The aim of the study is to determine the radon concentrations in the University Hospital of Bari, Apulia Region, Southern Italy. The monitoring took place from 2017 to 2018 for a total of 402 days and included 3492 premises. Radon environmental sampling was performed with passive dosimeters of the CR-39 type. The highest mean concentration was found in basement rooms (118.9 Bq/m^3^), followed by ground-floor rooms (88.2 Bq/m^3^), first-floor rooms (78.1 Bq/m^3^), second-floor rooms (66.7 Bq/m^3^), and third-floor rooms (68.9 Bq/m^3^). An average radon concentration lower than the WHO recommended level of 100 Bq/m^3^ was detected in 73.5% of monitored environments, while only 0.9% exceeded the reference level of 300 Bq/m^3^ set by the national law (Legislative Decree 101/2020). The frequency of environments in which radon concentrations exceed 300 Bq/m^3^ is significantly higher in the basement (*p*-value < 0.001). As for a previous preliminary investigation in the same hospital conducted on a much smaller number of premises (*n* = 401), most of the monitored environments had radon concentrations lower than the reference levels set by the new national law, and the risk to the healthcare workers’ health derived from occupational exposure to radon could be considered acceptable.

## 1. Introduction

Radon (222Rn) is a naturally occuring radioactive noble gas with an atomic number of 86 that is chemically and electrically neutral and ubiquitously widespread since it is generated by radium (226Ra), a decay product of uranium (238U), present in all rocks and soils. In particular, the major source of radon is generally attributed to rocks rather than soils; nevertheless, water and building materials, such as argil and concrete, also represent significant secondary sources. It is undetectable by sight, smell, or taste, is soluble in water, and has a density equal to 7.5 times that of atmospheric air. Among all the multiple environmental xenobiotics that pose a public health problem, radon is the largest source of natural background radiation to which the general population may be exposed and represents the primary source of annual effective radiation from all sources [1,2,3].

222Rn has a half-life of 3.8 days and decays by emitting ionizing radiation of the alpha type and forming its so-called decay products, or “progeny,” which also emit alpha radiation. These isotopes tend to move away from the initial site to escape into the atmosphere and be breathed by humans. In general, the concentrations of radon in outdoor air (outdoor radon) are very low; conversely, when a building is present on the ground, radon can penetrate and remain, reaching high concentrations (indoor radon). For this reason, radon is a risk factor in confined living and working environments such as public buildings (schools, hospitals) and recreational environments (cinemas, gyms, etc.) [4,5,6]. The geological nature of the site, the construction typology of the building, the construction materials used, and the ventilation methods are among the most determining parameters for the concentration of indoor radon [7].

Since 1988, radon has been classified as a proven carcinogen of Group 1 by the International Agency for Research on Cancer (IARC): breathing radon over time increases the risk of lung cancer [8,9,10,11]. Radon is the second leading cause of lung cancer in the United States, and the U.S. Environmental Protection Agency (EPA) estimates that about 21,000 people die every year from radon-related lung cancer [12]. Only smoking causes more lung cancer deaths [13]. Nevertheless, occupational exposure to high concentrations of radon also increases the risk of lung cancer in non-smokers [14]. As for asbestos-induced cancers, several studies are investigating the genetic susceptibility to the development of radon-related lung cancer [15,16].

Alternatively, the relationship between radon and other types of cancer, such as leukemia and Hodgkin lymphoma, has not been consistently demonstrated and needs further investigation [14,17,18].

The recommended radon levels, according to the World Health Organization (WHO) and the International Commission for Radiological Protection (ICRP), are set at a range of 100–300 Bq/m^3^ for residential spaces. According to these organizations, the concentration of radon in indoor air should not exceed 300 Bq/m^3^, in order to reduce the health risk to the lowest possible level [19,20].

In Italy, the recent Legislative Decree 101/2020 has brought greater attention to the issue of ionizing radiation exposure, including radon, by transposing the Basic Safety Standards Directive-2013/59/Euratom [21,22]. This directive replaces the former “national action level” of 500 Bq/m^3^ with a new “reference level” of 300 Bq/m^3^ for both homes and workplaces [23]. Employers have a legal obligation to regularly assess their employees’ potential exposure to radon, particularly in high-risk underground work environments such as caves, tunnels, underground parking garages, basements, and ground-floor workplaces located in areas with a high risk of radon exposure as identified by the National Radon Action Plan [24,25].

Furthermore, of all workplaces, constantly assessing radon levels in hospital settings is of the utmost importance. This is essential, as both patients and healthcare workers are exposed to health risks resulting from chronic radon exposure. In this regard, in 2014–2015, a study was conducted at the University Hospital of Bari, Apulia, southern Italy, to determine the radon concentrations in 401 premises of 28 different buildings of the hospital. Results showed that most workplaces (76.1%) at the university hospital reported radon concentrations within both the WHO recommended levels and those set by the regional law [26]. However, the measurements were carried out on a small number of premises, considering the entire hospital.

The aim of the study is therefore to carry out new measurements of the radon concentration in a larger number of the same university hospital working environments and compare the results with the different recommended and reference levels, including those established by the new national law, Legislative Decree 101/2020.

## 2. Materials and Methods

The study was conducted on the workplaces of the University Hospital of Bari, Apulia, southern Italy, which occupies an area of around 230 thousand square meters. The monitoring took place from 2017 to 2018 for a total of 402 days and included 3492 premises within approximately 30 different buildings. Among these, there were 755 basement rooms located roughly 3.5 m below street level, 1061 ground-floor rooms, 802 first-floor rooms, 588 s floor rooms, and 286 third-floor rooms. Each of the examined environments is continually inhabited by workers for a minimum of 6 h per day and 5 days per week. For each sample, a card was completed featuring the dosimeter code, the date of placement, the date of collection, and the identification of the local measurement object.

Environmental radon measurements were collected for two consecutive six-month periods: the first period (June–October) and the second period (October–June). For each floor, the average of the concentrations detected in each environment was calculated.

Although the assessment of radon levels was conducted in a workplace, we have chosen to compare the results not only with Italian law 101/2020 reference levels for workplaces but also with more stringent recommended levels, such as those for the general population, in the awareness that it is not possible to recognize any safe level of radon exposure [27]. Therefore, the results of the radon detection were interpreted based on recommended levels established by the World Health Organization (ranging from 100 to 300 Bq/m^3^), the Environmental Protection Agency action level (148 Bq/m^3^), and Italian law 101/2020 reference levels for workplaces (300 Bq/m^3^). Accordingly, indoor radon concentrations were divided into <100 Bq/m^3^, 100–148 Bq/m^3^, 149–300 Bq/m^3^, and >300 Bq/m^3^.

Environmental monitoring was carried out using CR-39 trace dosimeters. CR-39 is a passive type dosimeter, as it does not require an electrical power supply. It consists of a plastic frame that encloses an alpha particle detector made of a transparent allyl polymer known as CR-39. The positioning of the dosimeters was carried out taking into account the need to eliminate the effect of heat sources (i.e., radiators and windows): approximately 2 m above the ground and at least 0.3 m from the external walls. Radon penetrates these devices by diffusion or permeation and produces, as a result of its decay, a series of alpha radiations. These radiations ionize the solid matrix of the detector and produce so-called “nuclear tracks” due to the breaking of atomic bonds along a straight path. The density of the nuclear tracks reflects the radon concentration in the air (Bq/m^3^). The CR39 calibration constant was 0.41 Bq/m^3^ (1 track/cm^2^ of exposure), and the minimum measurable concentration for an exposure period of three months was 10 Bq/m^3^. All dosimeters were calibrated according to the same method as the previous study [26].

All collected data was reported in Excel (Microsoft Office, Microsoft Corporation, Redmond, WA, USA). Statistical analysis was performed using the ANOVA and Tukey’s post hoc tests (comparisons between means) and the chi-square test (comparison of categorical variables formulated in absolute number and percentage). Mean and standard deviation were used for qualitative and normally distributed quantitative variables. IBM SPSS Statistics Version 26 (SPSS Inc., Chicago, IL, USA, 2019) was the software of choice for data analysis. Statistical significance was set at *p*-value < 0.05.

## 3. Results

The mean annual concentration of radon in the hospital workplace environments varied significantly among the different floors (Table 1). The highest mean concentration was found in basement rooms (118.9 Bq/m^3^), followed by ground-floor rooms (88.2 Bq/m^3^), first-floor rooms (78.1 Bq/m^3^), second-floor rooms (66.7 Bq/m^3^), and third-floor rooms (68.9 Bq/m^3^).

The Tukey test showed significant variations in mean radon concentrations when comparing floors to each other. The comparison between the basement and the other floors (ground, first, second, and third) showed a strong statistical significance (*p*-value < 0.001), indicating a significantly higher radon concentration in the basement when compared with the other floors. The comparison between the first and second floors showed a strong statistical significance too (*p*-value < 0.001), revealing a higher radon concentration on the first floor when compared with the second floor, and the comparison between the first and third floors showed a small but still statistically significant difference in radon concentrations (*p*-value = 0.046). Finally, the comparison between the second and third floors showed a *p*-value of 0.970, suggesting no significant difference in radon concentrations between these two floors. In summary, this result highlighted that the concentration of radon gas in the hospital workplace environments is consistently higher in basement rooms and decreases as the floor level increases, with the exception of a possible slight increase from the first to the third floor.

During the 402 days of monitoring, an average radon concentration lower than the WHO recommended level of 100 Bq/m^3^ was detected in 73.5% of monitored environments, while only 0.9% exceeded the reference level of 300 Bq/m^3^ set by the national law (Legislative Decree 101/2020). In the 8.3% of rooms was found a radon concentration between 149 and 300 Bq/m^3^ (Table 2).

Analysis of radon levels for each floor (Table 3) showed that the frequency of environments in which radon concentrations exceed 300 Bq/m^3^ is significantly higher in the basement than in the other floors (*p*-value < 0.001). In particular, in twenty-six rooms of the basement, the recorded radon level was higher than 300 Bq/m^3^ (3.5% of the total basement rooms), as compared with only four ground-floor rooms (0.4% of the total ground-floor rooms) and only one first-floor room (0.1% of the total first-floor rooms).

## 4. Discussion

Our study aimed to evaluate the concentration of radon in the University Hospital of Bari and implement mitigation actions.

The monitoring covered almost 3500 hospital rooms or premises including patient rooms, ambulatory, operating rooms, offices, and environments in underground floors such as laboratories, storehouses, and archives. Most of the measurements carried out attested to low radon levels, and 99% of the premises studied showed a radon concentration lower than the reference level of 300 Bq/m^3^.

Although, as is known, it is currently not possible to recognize any safe level of radon exposure, these results allow us to assume that the health risk to workers from occupational radon exposure is low [27].

As expected, the concentration of radon gas in the hospital workplace environments was consistently higher in basement rooms and decreased as the floor level increased. It is important to emphasize that the basements of the hospital facilities are mainly represented by laboratories and warehouses, and there are no patient rooms located in the underground floors. This finding is in line with those reported in the literature and is due to the well-known natural movement of radon gas through the soil into the building, known as the “stack effect” [28,29,30]. The stack effect is the natural movement of air coming in and out of buildings, chimneys, and flue stacks: warm air inside the building rises, and cold air enters through the lower levels of the building, causing an accumulation of radon gas in those areas [31]. Additionally, high moisture levels in underground rooms due to poor ventilation can also contribute to the accumulation of radon, as the gas is more soluble in water than in air.

It is also interesting to compare these results with those of the study previously carried out in the same hospital. In 2014–2015, an average annual radon concentration of 48.0 Bq/m^3^ was found, and the average concentration was lower than 100 Bq/m^3^ in 76.1% of the premises studied, while it was higher than 300 Bq/m^3^ in the other 0.9% [26]. In the present study, the new measurements show that the percentage of rooms with values below 100 Bq/m^3^ is slightly lower (73.5%). However, this kind of comparison is affected by several biases since, at the time, the measurements were carried out on a much smaller number of premises (401 vs. 3492) on the lower floors only.

In recent decades in Italy, the National Institute of Health (ISS) and the actual Higher Institute for Environmental Protection and Research (ISPRA) conducted a survey to evaluate the indoor exposure of the Italian population, measuring radon concentrations in 5000 homes in 232 cities [32]. Although not recent (1989–1997), it remains the largest survey carried out up to now at a national level. An average radon concentration of 77 Bq/m^3^ emerged, and for the Apulia Region, an average concentration of 52 Bq/m^3^. Moreover, more recent measurements have documented, in Italy, an average radon concentration of 70 Bq/m^3^ [33]. Therefore, the radon levels in the Apulia Region can be considered to be in the medium range. Other studies in the region investigated the correlation between an increased incidence of lung cancer and chronic exposure to radon [34,35,36]. The results of these studies confirm that indoor radon pollution is a significant concern throughout the whole region and is one of the primary causes of lung cancer. Therefore, it is highly recommended to implement precautionary and mitigating measures to minimize exposure to indoor radon as much as possible.

The methods used to reduce radon levels in a building are different based on factors such as the desired level of reduction and the floor characteristics. The operating principles of the mitigation techniques are based on the reduction of the entry of radon from the ground into the building by means of various systems, used individually or in combination; it is also advisable to proceed gradually, carrying out first the interventions that are less expensive and invasive, to evaluate whether these alone can be decisive. The systems are based on the sealing of the access ways, on the natural or forced ventilation of the internal environments, or on the depressurization of the soil under the building [37].

Our study had certain limitations. First, it was not possible to analyze several factors influencing the radon concentration, such as the size and use of the rooms, the height between the floors, and the flow rate of the air circulation. Second, we were unable to measure the individual exposure to radon due to operational and logistical reasons. It would therefore be advisable for future studies to focus on this aspect. Despite these limitations, our work also has strengths. Compared to the previous study conducted in the same hospital, we have increased the number of rooms monitored by about 10 times (from 401 to 3492), which are furthermore distributed on several different levels. This allowed us to draw stronger conclusions about the radon exposure levels of healthcare workers and to analyze the spatial distribution of radon. Moreover, there are few studies published in the literature on hospital radon monitoring in such a large number of rooms distributed on different floors of a healthcare setting. Therefore, the results of this work reinforce the knowledge gained from the study of the spatial distribution of radon with the perspective of being able to measure the levels of radon throughout the national territory with a uniform measurement method in order to make the data of different surveys on the same territory comparable.

## 5. Conclusions

As for the previous preliminary investigation, most of the monitored environments (99%) at the University Hospital of Bari had radon concentrations lower than the reference levels, and the risk to the healthcare workers’ health derived from occupational exposure to radon could be considered admissible according to the new national law, Legislative Decree 101/2020. Nevertheless, the concentration of radon in hospital workplace environments is consistently higher in basement rooms, highlighting the need to reduce the use of underground floors for prolonged work activities, at least where structural improvements cannot be made. In environments where instead it is possible to implement structural improvements, a restructuring of the work environment with improvements to ventilation or the implementation of other mitigation methods becomes necessary, in light also of the scientific evidence showing that there is no safe radon level and in line with the mission to achieve as low as reasonably possible radon exposures to protect workers and the public.

## Figures and Tables

**Table 1:** Annual radon concentration in the different floors (Bq/m^3^).

Basement (n. 755 Rooms)		Ground (n. 1074 Rooms)		First (n. 797 Rooms)		Second (n. 585 Rooms)		Third (n. 276 Rooms)	
Mean	S.D.	Mean	S.D.	Mean	S.D.	Mean	S.D.	Mean	S.D.
118.9	74.1	88.2	44.1	78.1	38.7	66.7	21.6	68.9	30.4

**Table 2:** Levels of radon in relation to the World Health Organization (WHO) recommended level (100 Bq/m^3^), Environmental Protection Agency (EPA) action level (148 Bq/m^3^) and national law—Legislative Decree 101/2020 reference level (300 Bq/m^3^).

	Total Rooms (n. 3487)	
	n.	%
<100 Bq/m^3^	2581	73.5
100–148 Bq/m^3^	579	16.5
149–300 Bq/m^3^	291	8.3
>300 Bq/m^3^	30	0.9

**Table 3:** Levels of radon for each floor, in relation to the World Health Organization (WHO) recommended level (100 Bq/m^3^), Environmental Protection Agency (EPA) action level (148 Bq/m^3^) and national law—Legislative Decree 101/2020 reference level (300 Bq/m^3^).

	Basement (n. 755 Roms)		Ground (n. 1074 Rooms)		First (n. 797 Rooms)		Second (n. 585 Rooms)		Third (n. 276 Rooms)	
	n.	%	n.	%	n.	%	n.	%	n.	%
<100 Bq/m^3^	381	50.9	780	72.6	642	80.6	543	92.8	235	85.1
100–148 Bq/m^3^	195	26.0	196	18.2	114	14.3	39	6.7	35	12.7
149–300 Bq/m^3^	147	19.6	94	8.8	40	5.0	3	0.5	6	2.2
>300 Bq/m^3^	26	3.5	4	0.4	1	0.1	0	0	0	0

## Data Availability

The data presented in this study are available on request from the corresponding author.

## References

[1] United Nations (2010). Sources and Effects of Ionizing Radiation: United Nations Scientific Committee on the Effects of Atomic Radiation: UNSCEAR 2008 Report to the General Assembly, with Scientific Annexes. Scientific Committee on the Effects of Atomic Radiation.

[2] Vimercati L., Gatti M.F., Baldassarre A., Nettis E., Favia N., Palma M., Martina G.L., Di Leo E., Musti M. (2015). Occupational Exposure to Urban Air Pollution and Allergic Diseases. Int. J. Environ. Res. Public Health.

[3] Vimercati L. (2011). Traffic related air pollution and respiratory morbidity. Lung. India.

[4] Parsons J. (2002). Radon: A Homeowner’s Guide to Detection and Control.

[5] Garzillo C., Pugliese M., Loffredo F., Quarto M. (2017). Indoor radon exposure and lung cancer risk: A meta-analysis of case-control studies. Transl. Cancer Res..

[6] Krewski D., Lubin J.H., Zielinski J.M., Alavanja M., Catalan V.S., Field R.W., Klotz J.B., Létourneau E.G., Lynch C.F., Lyon J.L. (2006). A combined analysis of North American case–control studies of residential radon and lung cancer. J. Toxicol. Environ. Health A.

[7] WHO (2009). WHO Handbook on Indoor Radon: A Public Health Perspective.

[8] IARC (1988). IARC Monographs on the Evaluation of Carcinogenic Risks to Humans.

[9] Durante M., Grossi G., Pugliese M., Manti L., Nappo M., Gialanella G. (1994). Single charged-particle damage to living cells: A new method based on track-etch detectors. Nucl. Instrum. Methods Phys. Res. Sect. B Beam Interact. Mater. At..

[10] Lorenzo-González M., Torres-Durán M., Barbosa-Lorenzo R., Provencio-Pulla M., Barros-Dios J.M., Ruano-Ravina A. (2019). Radon exposure: A major cause of lung cancer. Expert Rev. Respir. Med..

[11] International Agency for Research on Cancer (IARC) (1988). Man-made Mineral Fibres and Radon. IARC Monographs on the Evaluation of Carcinogenic Risks to Humans.

[12] United States Environmental Protection Agency (EPA) A Citizen’s Guide to Radon. https://www.epa.gov/sites/default/files/2016-02/documents/2012_a_citizens_guide_to_radon.pdf.

[13] United States Environmental Protection Agency (EPA) What Is Radon Gas? Is It Dangerous?. https://www.epa.gov/radiation/what-radon-gas-it-dangerous.

[14] Al-Zoughool M., Krewski D. (2009). Health effects of radon: A review of the literature. Int. J. Radiat. Biol..

[15] Rosenberger A., Hung R.J., Christiani D.C., Caporaso N.E., Liu G., Bojesen S.E., Le Marchand L., Haiman C.A., Albanes D., Aldrich M.C. (2018). Genetic modifiers of radon-induced lung cancer risk: A genome-wide interaction study in former uranium miners. Int. Arch. Occup. Environ. Health.

[16] Serio G., Vimercati L., Pennella A., Gentile M., Cavone D., Buonadonna A.L., Scattone A., Fortarezza F., De Palma A., Marzullo A. (2018). Genomic changes of chromosomes 8p23.1 and 1q21: Novel mutations in malignant mesothelioma. Lung. Cancer.

[17] Ruano-Ravina A., Aragonés N., Kelsey K.T., Pérez-Ríos M., Piñeiro-Lamas M., López-Abente G. (2017). Residential radon exposure and brain cancer: An ecological study in a radon prone area (Galicia, Spain). Sci. Rep..

[18] Kendall G.M., Little M.P., Wakeford R. (2021). A review of studies of childhood cancer and natural background radiation. Int. J. Radiat. Biol..

[19] Valentin J. (2007). The 2007 Recommendations of the International Commission on Radiological Protection.

[20] Zeeb H., Shannoum F. (2009). WHO Guidelines Approved by the Guidelines Review Committee. WHO Handbook on Indoor Radon: A Public Health Perspective.

[21] Italian Governement (2020). Decreto Legislativo n. 101 del 31 Luglio 2020, Attuazione della Direttiva 2013/59/Euratom, che Stabilisce Norme Fondamentali di Sicurezza Relative alla Protezione Contro i Pericoli Derivanti Dall’esposizione alle Radiazioni Ionizzanti, e che Abroga le Direttive 89/618/Euratom, 90/641/Euratom, 96/29/Euratom, 97/43/Euratom e 2003/122/Euratom e Riordino della Normativa di Settore in Attuazione Dell’articolo 20, Comma 1, Lettera a, della Legge 4 Ottobre 2019, n. 117.

[22] European Council Directive (2014). 2013/59/Euratom on basic safety standards for protection against the dangers arising from exposure to ionising radiation and repealing Directives 89/618/Euratom, 90/641/Euratom, 96/29/Euratom, 97/43/Euratom and 2003/122/Euratom. Off. J. Eur. Union..

[23] Italian Governement (2000). Decreto Legislativo n. 241 del 26 maggio 2000, Attuazione della direttiva 96/29/EURATOM in Materia di Protezione Sanitaria della Popolazione e dei Lavoratori Contro i Rischi Derivanti Dalle Radiazioni Ionizzanti. Official Gazette No. 203. https://www.gazzettaufficiale.it/eli/id/2000/08/31/000G0271/sg.

[24] D’Avino V., Pugliese M. (2021). Radon Survey in Bank Buildings of Campania Region According to the Italian Transposition of Euratom 59/2013. Life.

[25] Italian Higher Institute of Health Protezione dal Radon. https://www.iss.it/protezione-dal-radon/-/asset_publisher/L6pCAKa9c9V6/content/protezione-dal-radon-1.

[26] Vimercati L., Fucilli F., Cavone D., De Maria L., Birtolo F., Ferri G.M., Soleo L., Lovreglio P. (2018). Radon Levels in Indoor Environments of the University Hospital in Bari-Apulia Region Southern Italy. Int. J. Environ. Res. Public Health.

[27] National Research Council (US) (1999). Committee on Health Risks of Exposure to Radon (BEIR VI).

[28] Afolabi O.T., Esan D.T., Banjoko B., Fajewonyomi B.A., Tobih J.E., Olubodun B.B. (2015). Radon level in a Nigerian University Campus. BMC Res. Notes.

[29] Kim S.H., Hwang W.J., Cho J.S., Kang D.R. (2016). Attributable risk of lung cancer deaths due to indoor radon exposure. Ann. Occup. Environ. Med..

[30] Cammarota B., Cascone M.T., De Paola L., Schillirò F., Del Prete U. (2009). Il rischio radon in ambienti sanitari: Monitoraggio ambientale e dose efficace [Radon risk in healthcare facilities: Environmental monitoring and effective dose]. Med. Lav..

[31] Boardman C.R., Glass S.V. (2015). Basement radon entry and stack driven moisture infiltration reduced by active soil depressurization. Build. Environ..

[32] Bochicchio F., Campos Venuti G., Piermattei S., Torri G., Nuccetelli C., Risica S., Tommasino L. Results of the National Survey on Radon Indoors in the all the 21 Italian Regions. Proceedings of the Radon in the Living Environment Workshop, Atene. Aprile 1999—APAT. Annuario dei dati ambientali, Concentrazione di Attività di Radon Indoor, Estratto Edizione 2005–2006.

[33] Bochicchio F., Campos Venuti G., Piermattei S., Nuccetelli C., Risica S., Tommasino L., Torri G., Magnoni M., Agnesod G., Sgorbati G. (2005). Annual average and seasonal variations of residential radon concentration for all Italian Regions. Radiat. Meas..

[34] Maggiore G., De Filippis G., Totaro T., Tamborino B., Idolo A., Serio F., Castorini I.F., Valenzano B., Riccio A., Miani A. (2020). Evaluation of radon exposure risk and lung cancer incidence/mortality in South-eastern Italy. J. Prev. Med. Hyg..

[35] Vimercati L., Cavone D., Delfino M.C., De Maria L., Caputi A., Sponselli S., Corrado V., Bruno V., Spalluto G., Eranio G. (2021). Relationships among Indoor Radon, Earthquake Magnitude Data and Lung Cancer Risks in a Residential Building of an Apulian Town (Southern Italy). Atmosphere.

[36] L’Abbate N., Marcuccio P., Dipace C., Carbonara M., Carioggia E., Martucci V., Salamanna S., Simeone G., Vitucci L. (2002). Contaminazione da radon indoor nelle abitazioni pugliesi e valutazione della probabilità di insorgenza di tumore polmonare nella popolazione residente [Indoor radon pollution in houses in the Apulian Region of Italy and evaluation of the probability of lung cancer in the population]. Med. Lav..

[37] Khan S.M., Gomes J., Krewski D.R. (2019). Radon interventions around the globe: A systematic review. Heliyon.

